# Nurses’ duty to care during the COVID-19 pandemic: a cross-sectional survey

**DOI:** 10.1186/s12912-022-01064-0

**Published:** 2022-11-02

**Authors:** Hyerine Shin, Kyung hee Kim, Ji-su Kim, Yeun-hee Kwak

**Affiliations:** Department of Nursing, Chung-Ang University, 84 Heukseok-ro Dongjak-Gu, 06974 Seoul, Republic of Korea

**Keywords:** Disaster nursing, Duty to care, Nursing ethics, Disaster planning, Surge capacity

## Abstract

**Background:**

Despite the increased demand for nurses worldwide, discussion of nurses’ duty to care is lacking. This study aimed to examine nurses’ duty to care during the coronavirus disease 2019 (COVID-19) pandemic and to identify the influencing factors.

**Methods:**

This was a cross-sectional descriptive research study that used a structured online questionnaire. Registered Korean nurses answered a demographic questionnaire and the Nash Duty to Care Scale.

**Results:**

Age and employment at tertiary hospitals increased nurses’ duty to care. Male sex, a highly educated status, and employment at tertiary hospitals increased the perceived risk. Male sex and employment at tertiary or general hospitals increased confidence in the employer, while a high level of education and a longer total clinical career decreased the same. Age and a higher monthly wage increased perceived obligation. Age, lack of religious beliefs, and clinical experience of 3–7 years increased professional preparedness.

**Conclusion:**

Without enough nursing manpower, the disaster response system could prove to be inefficient. Considering that adequate nurse staffing is essential in disaster management, it is crucial to ensure that nurses have a will to provide care in the case of disaster. In the future, a more active discussion on nurses’ duty to care and additional research on factors that may hinder and facilitate the same are needed.

## Background

The coronavirus disease 2019 (COVID-19) pandemic has highlighted an ethical issue concerning healthcare workers. Between February 12, 2020, and April 9, 2020, 19% of 315,531 confirmed COVID-19 cases were among healthcare workers [1]. A study in the UK and the US reported that the positive rate of COVID-19 frontline workers increased 12 times compared with that in the general community [2].

Frontline healthcare workers at the forefront of disaster situations take various risks in a highly strained environment to provide care to patients [3]. Due to its global shortage from the early stages of COVID-19, inadequate reuse of personal protective equipment (PPE) still poses a threat of infection among healthcare workers and to the stability of the healthcare system [4–6]. Moreover, healthcare workers are suffering from constant psychological stress and severe burnout due to the rapidly increasing workload, fatigue and fear from co-workers’ COVID-19 infections, and concerns about loved ones getting infected [7, 8]. Healthcare workers are often stigmatized as a source of infection by the community, ostracized, or even attacked [9, 10]. These various difficulties decrease the will to work among medical personnel [11] and increase their intention to leave their organizations [12]. This suggests that medical personnel are faced with various ethical conflicts during disasters, and social attention is needed for them.

The concept of duty to care encompasses ethical aspects of providing care to patients even in situations where healthcare providers might be at risk themselves [13]. In the past, a social contract model was used to define duty to care [14]. This implied that healthcare workers take a certain degree of personal risk because they may have certain privileges (such as financial rewards or the respect of society) in return for their roles. However, this view raises the ethical issue of how much healthcare workers must make sacrifices to provide care. Sokol emphasized the necessity of discussing the limits of duty to care and argued that it cannot be enforced in situations beyond the limits drawn through social consensus [15]. In modern times, the limits of the duty to care have been discussed [16–18].

Nurses are the largest expert group of healthcare workers [19]. As a front-line group, they should respond to disaster victims who need medical services [20]. Although the demand for nursing care in disaster situations has also grown due to the swell of disasters worldwide, there is a lack of discussion on nurses’ duty to care in disasters to date.

Previous research has focused on the construct of “duty to treat,” which encompasses all medical, healthcare, and first-aid workers. Moreover, studies on duty to treat confirmed medical personnel’s’ “willingness to work” by focusing on the availability of the medical system rather than on duty to treat itself. Most research on nurses’ duty to care has been limited to confirming the concept through literature reviews or examining the perceptions of medical personnel who provide nursing and medical care in disaster situations through qualitative research.

Therefore, this study aims to bridge this gap by quantitatively analyzing the level of clinical nurses’ duty to care during the COVID-19 crisis using the Nash Duty to Care Scale (NDCS) and to identify the factors influencing the same.

## Objective

The aims of the present study were to identify the following with regard to nurses’ duty to care during the COVID-19 pandemic: (a) the extent of the overall and four subscales of duty to care and (b) the differences in and influence of nurses’ demographic characteristics on duty to care.

## Methods

### Design

This cross-sectional descriptive research study was conducted in 2021 and was reported according to the STROBE checklist.

### Research participants and data collection

Convenience sampling was used to recruit participants. Participants were registered Korean nurses currently working at a clinical nursing practice. Those who had resigned, took a leave of absence, or did not work in clinical nursing were excluded. In this study, due to the lack of previous studies, the effect size was set according to a rule of thumb and Cohen’s comment, and the power was set according to Spurlock’s recommendation [21, 22]. Using the G-power version 3.1.9.7 program and applying ANOVA with significance level (α) of 0.05, a power (1-β) of 0.95, and an effect size (r) of 0.25, [21, 22] the sample size was anticipated to be at least 280. Although measuring slightly different concepts, we considered a dropout rate of 20% with reference to similar studies [23]; therefore, 336 participants were targeted. After receiving approval from the Institutional Review Board of Chung-Ang University (1041078-202103-HRBM-080-01), an online survey was conducted from May 11, 2021, to May 16, 2021. Participants were recruited using the convenience sampling method; a link was posted in a banner advertisement on Nursescape (https://www.nurscape.net), which has a subscriber count of more than 330,000 Korean nurses. The purpose and method of the study were described in detail before the survey. Only those who voluntarily agreed to participate in the study completed the self-report questionnaire. A total of 336 nurses were recruited during the survey period. In the case of any omission of response, the questionnaire was discarded. After excluding 16 questionnaires with missing data, data from 320 nurses were analyzed.

### Data analysis

The collected data were analyzed using SPSS Statistics 25.0. The demographic characteristics of the participants were analyzed using descriptive statistics such as frequencies, percentages, means, and standard deviations. The nurses’ duty to care was analyzed using descriptive statistics such as means, standard deviations, and maximum and minimum values. Nurses’ duty to care according to demographic characteristics was analyzed by an independent t-test and one-way ANOVA, followed by a post hoc Duncan test. Stepwise multiple regression analysis was used to identify factors affecting nurses’ duty to care.

### Instruments

Nurses’ duty to care was measured using the NDCS [24]. The NDCS [24] includes 19 items across four subscales: perceived risk (seven items, Cronbach’s α of 0.91), confidence in employer (three items, Cronbach’s α of 0.81), perceived obligation (five items, Cronbach’s α of 0.83), and professional preparedness (four items, Cronbach’s α of 0.85). Participants responded on a five-point Likert scale (1 = strongly disagree; 5 = strongly agree). Negatively written items were calculated by converting them into inverse scores. The possible scores ranged from 19 to 95, with higher scores indicating higher willingness to respond in a disaster situation. Two nursing professors, one English professor, and two nurses, including researchers who are bilingual speakers fluent in Korean and English, translated the NDCS [24] into Korean and revised it. The original developer of the NDCS ensured that the translated scale retained its original meaning. Several parts of the translated version were modified based on the developer’s direction. The final version of this scale was used in this study after approval.

Participants responded to questions regarding sociodemographic characteristics such as age, sex, education, marital status, religion, total clinical career, job position, work unit, monthly wage, and type of hospital (size of hospital, type of department, severity of patients treated). These demographic characteristics were based on previous studies [25–27]. The total clinical career was classified into four stages based on the revised and supplemented Benner’s [28] model [29].

## Results

Participants were registered nurses from Korea with an average age of 31.87 years. Most participants were female, unmarried, bachelor’s degree holders, staff nurses, and not religious (Table 1).

**Table 1:** Differences in duty to care by demographic characteristics (N = 320)

Variables	Categories	n (%)	Total score		Perceived risk		Confidence in employer		Perceived obligation		Professional preparedness	
			**M ± SD**	**t or F(p) Duncan**	**M ± SD**	**t or F(p) Duncan**	**M ± SD**	**t or F(p) Duncan**	**M ± SD**	**t of F(p) Duncan**	**M ± SD**	**t or F(p) Duncan**
Age	20–29	160 (50)	61.16 ± 8.73	2.539	24.25 ± 4.32	1.322	8.51 ± 2.88	0.547	17.78 ± 3.45	**2.841**	10.61 ± 3.22	**5.964**
	30–39	113 (35.3)	62.53 ± 8.28	(0.057)	23.62 ± 4.05	(0.267)	8.60 ± 2.49	(0.65)	18.13 ± 3.09	**(0.038)**	12.18 ± 3.69	**(0.001)**
	40–49	29 (9.1)	63.41 ± 10.27		25.24 ± 4.04		8.83 ± 2.93		18.76 ± 2.81	**a < d**	10.59 ± 4.12	**a < b**
	50 above	18 (5.6)	66.56 ± 7.27		24.44 ± 4.00		9.33 ± 2.59		19.94 ± 3.04		12.83 ± 3.63	
Sex	Female	307 (95.9)	61.96 ± 8.72	1.813	24.03 ± 4.11	1.334	8.59 ± 2.74	0.964	18.08 ± 3.24	0.884	11.27 ± 3.58	0.457
	Male	13 (4.1)	66.62 ± 7.71	(0.070)	26.54 ± 5.41	(0.182)	9.38 ± 2.57	(0.335)	19.00 ± 4.20	(0.377)	11.69 ± 3.88	(0.647)
Education	Associate’s degree^a^	35 (10.9)	59.86 ± 11.03	1.356	22.63 ± 4.08	**3.175**	8.63 ± 2.82	0.143	17.51 ± 3.67	**4.258**	11.09 ± 3.84	0.339
	Baccalaureate^b^	251 (78.4)	62.23 ± 8.24	(0.266)	24.21 ± 4.15	**(0.043)**	8.65 ± 2.70	(0.867)	18.00 ± 3.21	**(0.015)**	11.37 ± 3.49	(0.713)
	Master’s degree or above^c^	34 (10.6)	63.91 ± 9.23		25.06 ± 4.33	**a < c**	8.38 ± 2.91		19.59 ± 3.04	**a, b < c**	10.88 ± 4.04	
Marital status	Single	241 (75.3)	61.86 ± 8.47	2.075	24.17 ± 4.18	1.689	8.52 ± 2.73	4.867	18.05 ± 3.37	0.077	11.12 ± 3.37	2.190
	Married	76 (23.8)	62.83 ± 9.47	(0.354)	23.91 ± 4.27	(0.430)	8.82 ± 2.72	(0.088)	18.33 ± 3.07	(0.962)	11.78 ± 4.12	(0.335)
	Other	3 (0.9)	68.33 ± 8.02		26.33 ± 2.31		11.67 ± 1.53		18.00 ± 1.00		12.33 ± 5.51	
Religion	Yes	119 (37.2)	62.04 ± 8.95	0.170	24.17 ± 4.54	0.131	8.92 ± 2.79	1.501	18.06 ± 3.42	0.238)	10.90 ± 3.57	1.494
	No	201 (62.8)	62.21 ± 8.60	(0.865)	24.10 ± 3.98	(0.896)	8.44 ± 2.70	(0.134)	18.15 ± 3.20	(0.812)	11.52 ± 3.58	(0.136)
Total clinical	< 1 ^a^	68 (21.3)	60.79 ± 8.83	1.263	24.04 ± 4.12	1.393	9.74 ± 2.94	**6.093**	17.34 ± 3.42	**3.486**	9.68 ± 3.13	**9.070**
career (years)	1–3 ^b^	65 (20.3)	62.11 ± 7.96	(0.287)	24.91 ± 4.01	(0.245)	8.23 ± 2.67	**(< 0.001)**	17.89 ± 3.25	**(0.016)**	11.08 ± 3.37	**(< 0.001)**
	4–6 ^c^	108 (33.8)	61.99 ± 8.44		23.59 ± 4.13		8.06 ± 2.35	**a > b, c**	18.07 ± 3.24	**a < d**	12.27 ± 3.39	**a < c, d**
	≥ 7 ^d^	79 (24.7)	63.57 ± 9.50		24.29 ± 4.43		8.75 ± 2.84		19.03 ± 3.07		11.51 ± 3.92	
Job position	Staff nurse^a^	286 (89.4)	61.74 ± 8.76	5.774	24.10 ± 4.20	1.149	8.56 ± 2.78	1.796	17.95 ± 3.32	**7.797**	11.13 ± 3.51	**7.516**
	Charge nurse^b^	21 (6.6)	65.38 ± 8.30	(0.056)	23.76 ± 4.48	(0.563)	9.38 ± 1.96	(0.407)	18.90 ± 2.21	**(0.020)**	13.33 ± 3.77	**(0.023)**
	Head nurse or above^c^	13 (4.1)	65.92 ± 6.59		25.31 ± 3.45		8.69 ± 2.75		20.46 ± 2.82	**a, b < c**	11.46 ± 4.10	**a < b**
Work unit	IM^a^	80 (25.0)	62.61 ± 8.79	1.174	23.89 ± 3.96	0.118	8.61 ± 2.45	0.361	17.68 ± 3.12	1.417	12.44 ± 3.31	**5.372**
	GS^b^	87 (27.2)	60.69 ± 8.61	(0.320)	24.18 ± 3.98	(0.949)	8.39 ± 2.76	(0.781)	17.84 ± 3.34	(0.238)	10.28 ± 3.60	**(0.001)**
	Special unit^c^	72 (22.5)	63.03 ± 8.43		24.21 ± 4.45		8.83 ± 2.81		18.56 ± 3.39		11.43 ± 3.22	**b < a**
	Others^d^	81 (25.3)	62.48 ± 8.97		24.23 ± 4.46		8.68 ± 2.92		18.46 ± 3.24		11.11 ± 3.85	
Monthly Wage	< 300	141 (44.1)	60.80 ± 8.63	**2.477**	23.62 ± 4.22	1.919	8.7 ± 2.91	0.484	17.47 ± 3.34	**3.179**	11.01 ± 3.70	1.243
	≥ 300	179 (55.9)	63.21 ± 8.66	**(0.014)**	24.53 ± 4.13	(0.056)	8.55 ± 2.59	(0.629)	18.63 ± 3.15	**(0.002)**	11.51 ± 3.48	-0.215
Type of	Tertiary hospital	151 (47.2)	63.02 ± 9.63	0.982	24.51 ± 4.12	2.819	8.18 ± 2.69	**9.595**	18.63 ± 3.08	1.858	11.69 ± 3.60	**8.200**
hospital	General Hospital	101 (31.6)	62.03 ± 8.14	(0.376)	24.34 ± 4.34	(0.061)	9.29 ± 2.76	**(< 0.001)**	17.89 ± 3.42	(0.158)	10.50 ± 3.34	**(< 0.001)**
	Other hospitals^e^	68 (21.3)	61.13 ± 8.51		23.07 ± 3.81		7.78 ± 2.36	**a > b, c**	17.84 ± 3.19		12.44 ± 3.72	**a < b, c**

^a^IM = Internal medicine; ^b^GS = General surgery; ^c^Special unit = Emergency room, intensive care unit, operating room; ^d^Others = All departments that are not internal medicine, general surgery, or special unit, such as psychiatry, neonatal room, delivery room, etc.; ^e^Other hospitals: Clinics, specialized hospitals, nursing hospitals, health care institutions, etc.

### Level of nurses’ duty to care during the COVID-19 pandemic

The nurses’ average score for duty to care was 62.15. The average scores on the subscales of perceived risk, confidence in employer, perceived obligation, and profession preparedness were 24.13, 8.62, 18.12 and 11.29, respectively. (Table 2)

**Table 2:** Level of nurses’ duty to care (N = 320)

Variables	Min	Max	M ± SD	Mean ± SD by item
Total Score	30.00	89.00	62.15 ± 8.71	3.20 ± 0.47
Perceived risk	12.00	35.00	24.13 ± 4.19	3.45 ± 0.60
Confidence in employer	3.00	15.00	8.62 ± 2.73	2.87 ± 0.91
Perceived obligation	8.00	25.00	18.12 ± 3.28	3.67 ± 0.67
Professional preparedness	4.00	20.00	11.29 ± 3.58	2.82 ± 0.90

### Differences in nurses’ duty to care by demographic characteristics

The results of the t-test and ANOVA indicated that the perceived risk was higher among nurses with a master’s degree or higher (F = 3.175) than among those who held a bachelor’s degree (F = 0.043). Confidence in the employer was significantly higher among nurses with less than one year of experience than among those with 1–7 years of experience (F = 6.093, p < 0.001). It was also significantly higher among nurses who worked at a tertiary general hospital than among those who worked at a general hospital or other hospitals (F = 9.595, p < 0.001; Table 1).

Perceived obligation showed significant differences based on education, total clinical career, job position, and monthly wage. Nurses with a master’s degree or higher demonstrated significantly higher scores on the confidence in employer dimension than those with an associate’s or bachelor’s degree (F = 4.258, p = 0.015). Participants with seven or more years of experience had significantly higher scores than those with less than one year of experience (F = 3.486, p = 0.016). In addition, nurses with a job position as head nurses or above (F = 7.797, p = 0.02) had higher scores than staff nurses (F = 7.797, p = 0.02; Table 1).

Professional preparedness showed significant differences based on total clinical career, job position, work unit, and type of hospital. Nurses with less than one year of experience reported lower professional preparedness than those with 3–7 years and seven or more years of experience (F = 9.07, p < 0.001). Nurses working in the internal medicine unit reported higher professional preparedness than those working in the general surgery unit (F = 5.372, p = 0.001). Those working at tertiary hospitals reported lower professional preparedness than those working in general hospitals and other hospitals (F = 8.200, p < 0.001). In the group of nurses with monthly wages of 3 million won or more, the total score for duty to care was significantly higher than that of nurses with montly wages of less than 3 million won (t = 2.477, p = 0.014; Table 1).

### Multiple regression analysis showing factors influencing nurses’ duty to care

To verify the factors affecting duty to care, a stepwise multiple regression analysis was performed by inputting demographic characteristics as independent variables. Duty to care (F = 3.514, p = 0.002) and its subscales revealed suitable regression models. There was no autocorrelation between each independent variable. As a result of examining the multicollinearity between the independent variables and the variance inflation factor (VIF), the values ​​for all variables ranged from a minimum value of 1.005 to a maximum value of 1.738 to less than 10, indicating that there was no problem with multicollinearity between the variables.

Factors influencing perceived risk were being male (β = 0.117, p = 0.035), holding a master’s degree or higher (β = 0.155, p = 0.04), and working at a tertiary hospital (β = 0.151, p = 0.039). Factors affecting confidence in the employer were older age (β = 0.315, p < 0.001), education status of a master’s degree or higher (β = 0.159, p = 0.040), 1–3 years of work experience (β = 0.289, p = 0.006), 3–7 years of work experience (β = 0.367, p < 0.001), seven or more years of work experience (β = 0.255, p = 0.008), employment at a tertiary hospital (β = 0.166, p = 0.014), and employment at a general hospital (β = 0.351, p < 0.001). Factors affecting perceived obligation were older age (β = 0.127, p = 0.024) and monthly wage of three million won or more (β = 0.151, p = 0.007). Factors influencing professional preparedness were older age (β = 0.231, p < 0.001), lack of religious beliefs (β = 0.126, p = 0.011), 3–7 years of work experience (β = 0.209, p = 0.004), employment as a charge nurse (β = 0.155, p = 0.008), employment in the general surgery unit (β = 0.194, p = 0.005), employment in other units (β = 0.160, p = 0.022), and employment at a tertiary hospital (β = 0.161, p = 0.037). Overall, the factor that influenced duty to care the most was older age (β = 0.235, p < 0.001; Table 3).

**Table 3:** Multiple regression analysis showing factors influencing duty to care (N = 320)

Variables	Categories	Total score			Perceived risk			Confidence in employer			Perceived obligation			Professional preparedness		
		**β**	**t**	***p***	**β**	**t**	***p***	**β**	**t**	***p***	**β**	**t**	***p***	**β**	**t**	***p***
Age		0.235	3.377	**< 0.001**				0.315	6.587	**< 0.001**	0.127	2.265	**0.024**	0.231	6.04	**< 0.001**
Sex	Female															
	Male				0.117	2.116	**0.035**									
Education	Associate’s degree^a^															
	Baccalaureate^b^				0.121	1.600	0.111	− 0.003	− 0.044	0.965						
	Master’s degree or above^c^				0.155	2.066	**0.04**	− 0.159	-2.060	**0.040**						
Religion	Yes															
	No													0.126	2.336	**0.011**
Total clinical	< 1 ^a^															
career	1–3 ^b^							− 0.289	-2.794	**0.006**				0.111	1.673	0.095
(years)	4–6 ^c^							− 0.367	-3.744	**< 0.001**				0.209	2.87	**0.004**
	≥ 7 ^d^							− 0.255	-2.688	**0.008**				− 0.016	− 0.173	0.863
Job position	Staff nurse															
	Charge nurse													0.155	2.678	**0.008**
	Head nurse or above													− 0.018	− 0.287	0.774
Work unit	IM^a^															
	GS^b^													− 0.194	-2.896	**0.005**
	Special unit^c^													− 0.048	− 0.725	0.369
	Others^d^													− 0.160	-2.307	**0.022**
Monthly	< 300										1					
Wage	≥ 300										0.151	2.707	**0.007**			
Type of	Tertiary hospital	0.172	2.271	0.024	0.151	2.079	**0.039**	0.166	2.261	**0.014**				− 0.078	-1.055	0.292
hospital	General hospital	0.118	1.491	0.137	0.114	1.541	0.124	0.351	4.502	**< 0.001**				− 0.161	-2.098	**0.037**
	Other hospitals^e^	1			1			1								
R^2^		0.045			0.030			0.112			0.040			0.145		
F (*p*)		3.514 (0.002)			2.989 (0.012)			6.048 (< 0.001)			7.685 (< 0.001)			5.514 (< 0.001)		

^a^IM = Internal medicine; ^b^GS= General surgery; ^c^Special unit = Emergency room, intensive care unit, operating room; ^d^Others= All departments that are not internal medicine, general surgery, or special unit, such as psychiatry, neonatal room, delivery room, etc.; ^e^Other hospitals: Clinics, specialized hospitals, nursing hospitals, health care institutions, etc.

In this study, the overall reliability of the tool was good, as indicated by Cronbach’s α of 0.78. A similar pattern was observed for the reliability of the subscales, with Cronbach’s α values of 0.73, 0.67, 0.71, and 0.88 for perceived risk, confidence in employer, perceived obgligation, and professional preparedness, respectively.

## Discussion

This study examines Korean nurses’ duty to care during the COVID-19 pandemic, the extent of the overall and four subscales of duty to care, and the influence of demographic characteristics on duty to care. Participants reported an average duty to care score of 62.15, which is lower than that of a study that measured duty to care among Taiwanese and US nurses [30]. In particular, the nurses’ scores on the subscales of confidence in employer, perceived obligation, and professional preparedness were lower than those of nurses in Taiwan and the US (Table 2). This may be because nurses in this study were working, directly or indirectly, for a prolonged period of the COVID-19 pandemic. At the beginning of the pandemic, the dedication displayed by nurses in Korea was lauded as a “heroic act.” However, adequate support was not provided, leading to most nurses working without education and training in disaster nursing [31]. Therefore, dissatisfaction with insufficient support and resources may have lowered Korean nurses’ duty to care. Duty to care increased with older age. This is consistent with the literature demonstrating that older nurses had increased willing to work in disaster situations [32].

Perceived risk measures the willingness to go to work in disaster situations despite being aware of the risk related to a disaster. Male nurses showed higher perceived risk than feamle nurses, which is consistent with the findings of previous studies [11, 33]; however, it is difficult to generalize this finding. Our study only included 13 male participants; thus, personal backgrounds such as having children or joining the military may have impacted the results. Perceived risk among nurses working in tertiary hospitals was higher than that among those working in other hospitals. Small- and medium-sized hospitals in Korea usually have much lower wages, poorer welfare, more chronic shortages of nurses, and more additional tasks such as guidance and supervision of non-professionals than tertiary hospitals [34]. Given that sufficient resources increase employers’ sense of calling [35, 36, 37], nurses in relatively affluent tertiary hospitals may be more inclined to take risks when disaster strikes.

Confidence in the employer measures the degree of trust in an organization with disaster response capabilities. Nurses’ confidence in the employer increased with age and with employment at tertiary hospital and showed a tendency to gradually decrease as their levels of education and experience increased. As nurses’ careers and educational backgrounds flourish, their expectations about their working environments and organizations grow. However, most work environments in hospitals are chronically under-supported, and these gaps can lead to dissatisfaction with the organization [34]. As support from an organization is associated with trust [35], dissatisfaction with the working environment and organization may lower confidence in the employer. In particular, a work experience level of 3–7 years had the greatest effect on confidence in employers. Nurses with 3–7 years of work experience are proficient in nursing practice, grow into professionals, and play the role of a preceptor to novice nurses [29]. Therefore, to improve duty to care among nurses, it is necessary to increase confidence in employers for competent nurses and to develop customized confidence in employer promotion programs by career.

Perceived obligation measures the willingness to go to work due to ethical and legal obligations, even in times of disaster. Perceived obligation increased as age and monthly wage increased. Perceived obligation is a concept similar to sense of calling [38], and sufficient compensation for work can increase job satisfaction and sense of calling [39–41]. Although there are many types of compensation, it seems that more detailed research on financial compensation and nurses’ perceived obligation is needed.

Professional preparedness measures the degree to which a person thinks that they have the ability, knowledge, and capacity to provide appropriate medical services in the event of a disaster. Professional preparedness tended to increase with age and work experience of three to seven years. These results are partially consistent with those of a previous study which state that with increased age and experience, various clinical careers can be built, which can have a positive effect on disaster nursing competency [42]. Nurses working in the general surgery unit and other units showed lower professional preparedness than those working in the internal medicine unit. Since COVID-19 is fundamentally a respiratory disease [43], nurses working in internal medicine units who are more familiar with respiratory diseases may feel a relatively high level of professional preparedness. In addition, charge nurses reported a higher level of professional preparedness. Charge nurses are practitioners in the intermediate stage between head and staff nurses and are involved in the work of both nursing practice and nursing management [42]. In the context of COVID-19, where manpower and resources were scarce, charge nurses may have been given more empowerment and autonomy than ever before. Since various studies have reported the contribution of empowerment and autonomy to individual competency and job performance [44–47], it is possible that the increased empowerment and autonomy of charge nurses may have resulted in increased professional preparedness.

Overall, the subdomains of duty to care, confidence in employer (Adjusted R^2^ = 0.112) and professional preparedness (Adjusted R^2^ = 0.145) were relatively well explained by sociodemographic factors. However, perceived risk (Adjusted R^2^ = 0.030) and perceived obligation (Adjusted R^2^ = 0.040) were relatively difficult to explain with demographic factors, suggesting that there are additional factors that impact these two domains.

This study has some limitations. First, as this study was conducted through an online survey, there may have been selection bias of users who use the Internet, thereby decreasing the generalizability of the findings. Second, due to the lack of prior research, only sociodemographic characteristics were analyzed to explain nurses’ duty to care. Third, the reliability of the subscale of confidence in employers was low (Cronbach’s α of 0.67). This is thought to be due to the cultural, social, and medical system differences between the US, where the tool was developed, and Korea.

In general, disaster response systems are built on the assumption that an adequate number of personnel will be deployed at the disaster site. If fewer staff members participate in disaster response, the safety, quality, and sustainability of medical services provided will be at risk [48]. The shortage of medical personnel not only increases mortality as a result of missing early warning signs in patients but also causes medical errors due to fatigue and increases the spread of infectious diseases [49]. In particular, since Florence Nightingale, nurses have been an essential professional group in responding to disasters and mass accidents [48, 50]. Therefore, adequate staffing of nurses in disaster situations is a key issue in disaster management. Furthermore, this study is the first to quantitatively analyze Korean nurses’ duty to care, which may be foundational for related studies in the future. Moreover, this study included nurses practicing in the clinic during the COVID-19 pandemic. This is meaningful as it served as an opportunity to understand duty to care among nurses close to the clinical field.

## Conclusion

To our knowledge, this is the first cross-sectional study to measure nurses’ duty to care through a structured survey and to analyze factors influencing the same during the COVID-19 pandemic. Although nurses play an important role in responding to disaster victims on the frontlines, they have been facing a large-scale pandemic without considering their duty to care. This has led to various ethical issues. Nurses’ willingness and effort to provide care in disaster situations are important components of the disaster response. Therefore, it is crucial to improve their duty to care through various interventions. These efforts must be as per social norms and policy, beyond the individual or organizational level. In the future, a more active discussion of nurses’ duty to care and additional research on factors that may hinder and improve the same are needed.

## Data Availability

The datasets generated and/or analysed during the current study are not publicly available due to legal restrictions imposed by the government of South Korea in relation to the “Personal Information Protection Act” but are available from the corresponding author on reasonable request.

## List of abbreviations

NDCS: Nash Duty to Care Scale
PPE: Personal protective equipment
